# Deficit of Omega-3 Fatty Acids in Acne Patients—A Cross-Sectional Pilot Study in a German Cohort

**DOI:** 10.3390/life14040519

**Published:** 2024-04-17

**Authors:** Anne Guertler, Tobias Fiedler, Diana Lill, Anne-Charlotte Kuna, Arina Volsky, Jens Wallmichrath, Till Kämmerer, Lars E. French, Markus Reinholz

**Affiliations:** 1 Department of Dermatology and Allergy, LMU University Hospital, 80539 Munich, Germanymarkus.reinholz@med.uni-muenchen.de (M.R.); 2 Dr. Phillip Frost Department of Dermatology and Cutaneous Surgery, Miller School of Medicine, University of Miami, Miami, FL 33136, USA

**Keywords:** clinical nutrition, nutrition, diet, oral supplementation, supplements, nutraceuticals

## Abstract

Omega-3 fatty acids (ω-3 FAs) exert anti-inflammatory effects, including the downregulation of pro-inflammatory cytokines, eicosanoids, and insulin-like growth factor-1. Therefore, they may improve acne severity as an adjunct treatment. However, there is a paucity of data regarding patients’ existing deficits. The aim of this study was to determine ω-3 FA levels in acne patients in correlation with self-reported dietary preferences and clinical severity. A single-center, cross-sectional study of 100 acne patients was conducted. Patients’ blood parameters, including ω-3 FAs levels, were assessed using the HS-omega-3 Index^®^ in erythrocytes (Omegametrix^®^ GmbH, Martinsried, Germany). Dietary preferences were assessed using a standardized food frequency questionnaire. Clinical dermatologic evaluation was performed using the Investigator Global Assessment (IGA) of acne. The values of the HS-omega-3 Index^®^ were outside the recommended range of 8–11% in 96 patients (mean 5.15%), independent of the clinical severity or affected anatomic sites. A severe deficit (HS-omega-3 Index^®^ < 4%) was seen more commonly in men than in women (*p* = 0.021). The regular consumption of legumes was significantly associated with higher ω-3 FA levels (*p* = 0.003), as was oral ω-3 FA supplementation (*p* = 0.006) and the lack of sunflower oil intake (*p* = 0.008). This pilot study demonstrated a deficit of ω-3 FAs in a German acne cohort. Higher ω-3 FAs levels were observed in patients with regular legume intake and oral ω-3 FAs supplementation. Further prospective studies are needed to investigate whether the clinical severity of acne improves in patients with normal HS-omega-3 Index^®^.

## 1. Introduction

Diet plays an important role in the prevention, onset, and course of disease, including dermatologic conditions such as acne vulgaris [1,2,3,4]. Any acne treatment should include a multifaceted approach aimed at addressing the main underlying causes of this skin condition (hormone-stimulated excess sebum production, irregular keratinisation, bacterial growth of *Cutibacterium acnes* (*C. acnes*), and inflammation) [5,6,7]. Pharmacological treatments include topical combinations (e.g., retinoids, benzoyl peroxide (BPO), or antibiotics) for mild to moderate acne and systemic options (e.g., retinoids or antibiotics) for moderate to severe cases [8]. Accompanying measures should always include a skin care regime targeted to individual needs, with daily use of sunscreen, optional (device) treatments (e.g., non-mechanical peelings or light therapy), and a consideration of exposome factors, including diet [9,10].

Recently, there has been a growing interest in identifying nutrients that may alleviate the severity of acne. Nutraceuticals, products derived from food sources that provide both nutritional and medicinal benefits, continue to be of particular interest for the management of skin diseases, including acne [11,12,13]. This interest has been fueled in part by patients themselves, most of whom are interested in natural and complementary therapies as either an adjunct or alternative to prescription medications [8,14,15]. However, given the wide range of dietary supplements available, their safety and efficacy must be carefully considered. Amongst currently available studies on nutraceuticals for acne, a recent systematic review has identified 42 placebo-controlled studies (with a total of 3346 participants) of fair or good quality, showing a potential benefit of Zinc, vitamin B_5_ and D, botanical extracts including green tea, probiotics, and Omega-3 (ω-3) fatty acids (FAs) in the treatment of acne [11].

**Figure 1:**
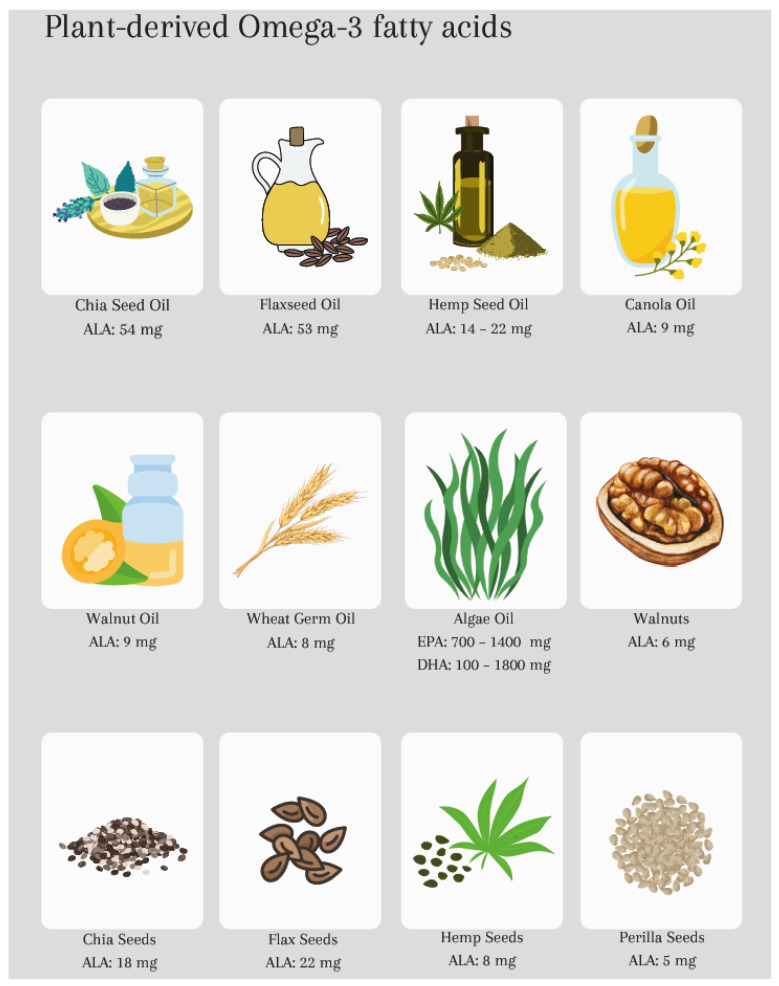

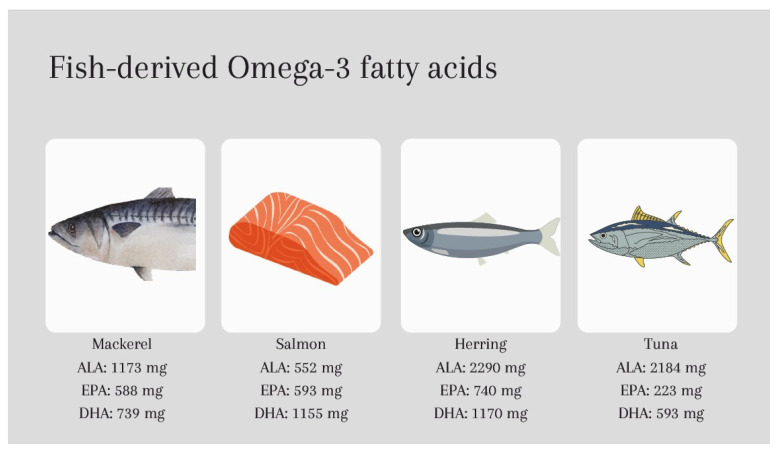
Approximate values of plant-derived and fish-derived omega-3 fatty acids (mg/100 g) in different food sources. Note: Exact content may vary depending on factors such as growing conditions and processing methods of nutrients.

ω-3 FAs are nutrients that cannot be synthesized by the human body and must therefore be obtained from the diet. They are so-called polyunsaturated FAs (PUFAs) because of the double bond in the third position of their fatty acid chain. There are plant- and algae/fish-derived sources. While plant-derived ω-3 FAs are primarily in the form of Alpha-linolenic acid (ALA), which must be converted by the body to the more biologically active forms of eicosapentaenoic acid (EPA) and docosahexaenoic acid (DHA), with conversion rates varying among individuals [16], algae/fish sources mainly contain EPA and DHA (Figure 1).

All humans have at least minimal levels of EPA and DHA, which have multiple physiological roles in the human body throughout life, and consequently diverse potential medical applications and therapeutic benefits. For example, ω-3 FAs are essential for the development of brain structure and function as they are an important component of cell membranes [17]. That is why recent studies have also emphasized their importance during pregnancy [18]. They are also thought to affect neurotransmitter function, which may help alleviate symptoms of depression and anxiety [19]. Benefits have also been reported for inflammatory conditions such as arthritis [20] and cardio-vascular health [21]. According to the European Food Safety Authority (EFSA), a daily intake of up to 5 g of combined EPA and DHA by oral supplementation is considered safe for adults, while the American Food and Drug Administration (FDA) has recommended a maximum of 3 g per day by oral supplementation [21,22,23]. Following a multicenter, double-blind, randomized, placebo-controlled trial in patients with cardiovascular disease or diabetes, the FDA and the European Medicines Agency (EMA) recently approved the use of 2 g of icosapent ethyl, a derivate of EPA, twice daily in adults with elevated triglyceride levels who were receiving statin therapy as an adjunct. According to the study, the risk of major ischemic events, including cardiovascular death, was significantly lower with a daily dose of 4 g icosapent ethyl than with placebo [24].

Recently, studies have evaluated the broad clinical implication of oral ω-3 FA supplementation in the prevention and management of skin diseases [25], most notably atopic dermatitis [26,27], psoriasis [28,29], and acne [30]. Overall, ω-3 FAs may reduce inflammation by stimulating the production of anti-inflammatory prostaglandin E1 (PGE_1_) and E3 (PGE_3_) as well as leukotriene B5 (LTB_5_) [31,32,33]. Furthermore, and especially in regard to acne, they may reduce levels of insulin-like growth factor (IGF)-1, the central nutrient inducer of acne [1,34]. IGF-1 activates the nutrient-sensitive kinase mammalian target of rapamycin complex-1 (mTORC1), which triggers anabolic pathways, including an increase in seborrhea and follicular hyperkeratosis [35]. Both are key factors in the pathogenesis of acne vulgaris [36]. Thus, ω-3 FAs can potentially lead to decreased sebum production and he keratinization of the pilosebaceous unit, thereby reducing the clinical severity of acne.

To date, three prospective interventional studies have been published evaluating the clinical effects of oral ω-3 FA supplementation in acne patients [30,31,37,38]. However, the data were inconclusive due to the limited study design, providing insufficient evidence to make clinical recommendations regarding potential benefits. In order to investigate the effect of supplementation, the baseline ω-3 FA levels of acne patients need to be assessed to determine whether there is a true deficit [30]. Currently, data on ω-3 FA blood levels in patients with acne vulgaris are lacking.

To fill this gap, the aim of the present study was to investigate the erythrocyte ω-3 FA blood levels in acne patients and to correlate them with the clinical severity of the disease and patients’ dietary habits. The results of this study may serve as the basis for future interventional trials.

## 2. Materials and Methods

### 2.1. Study Design

This single-center, cross-sectional study included 100 patients suffering from facial acne vulgaris of varying severity. All patients were recruited from the specialized outpatient clinic for acne at the Department of Dermatology and Allergy of the Ludwig Maximilian University (LMU) Munich, Germany. Inclusion criteria comprised healthy acne patients starting from their 12th year of age, irrespective of their clinical severity and current acne treatment. Healthy was defined as an absence of any prior medical condition. Written informed consent was obtained from all adult subjects involved in the study as well as from the respective parents or legal guardians of minors. Exclusion criteria were pregnancy and breastfeeding and missing consent. Ethical approval was obtained by the ethics committee of the medical faculty (Ref.-No. 20-0855). The study was performed in adherence with the Declaration of Helsinki (1996) and in accordance with regional laws and with the principles of Good Clinical Practice (GCP) for studies in human subjects. Patient information and identification were kept confidential and data analysis was performed anonymously.

### 2.2. Assessments and Outcomes

#### 2.2.1. Laboratory Analysis

Blood was drawn according to a standardized protocol, and a detailed laboratory analysis including levels of ω-3 FA was carried out using the HS-omega-3 Index^®^ (Omegametrix^®^ GmbH, Martinsried, Germany). Fatty acid methyl esters were generated from erythrocytes by means of acid transesterification and analyzed by means of gas chromatography using a GC2010 Gas Chromatograph (Shimadzu, Duisburg, Germany) equipped with a SP2560, 100 m column (Supelco, Bellefonte, PA, USA) using hydrogen as the carrier gas. Fatty acids were identified by comparison with a standard mixture of fatty acids characteristic of erythrocytes. Results were given as EPA plus DHA expressed as a percentage of a total of twenty-six specific fatty acids in erythrocytes after response factor correction. The coefficient of variation for EPA plus DHA was 5%. Analyses were quality-controlled according to DIN ISO 15189. The value accurately reflected the ω-3 FA status of a human individual [39]. The standardized laboratory procedure was robust to daily fluctuations and not influenced by severe clinical events. The HS-omega-3 Index^®^ target range was defined between 8 and 11%. A deficit was defined as <8%, while levels of <4% were defined as a severe deficit [22].

#### 2.2.2. Clinical Assessments

Acne vulgaris was classified by an independent dermatologist using the Global Investigator Assessment (IGA) [40]. Based on the count of non-inflammatory (comedones), and inflammatory lesions (papules and pustules), acne was divided into its three main subtypes: acne comedonica (IGA 1), acne papulopustolosa (IGA 2, 3), and acne conglobata (IGA 4) [41]. Patients’ height and weight were measured and the body mass index (BMI) was calculated (body weight in kilogram (kg)/height in meter (m)^2^): <18.5 underweight; 18.5–24.9 normal; 25.0–29.9 overweight; 30.0–34.9 obese; >35 extremely obese.

#### 2.2.3. Questionnaires

Basic demographic information and lifestyle aspects were obtained via a self-developed detailed questionnaire. Patients’ dietary habits over the previous four weeks were investigated using a standardized food frequency survey (FFS) comprising 125 detailed questions. The FFS provided insights into the overall intake and frequency of various foods and beverages [42,43]. The dermatology life quality index (DLQI) was used to evaluate the impact of acne on patients’ quality of life including psychological disability at the workplace, social and sexual relationships, depression, and anxiety. The higher the score, the more the patient’s quality of life was affected, with scores ranging from 0 to 30 (0–1 no effect, 2–5 small effect, 6–10 moderate effect, 11–20 very large effect, and 21–30 extremely large effect) [44].

### 2.3. Objectives

The primary objective of the study was to assess ω-3 FA blood levels in acne patients. Secondary objectives included evaluating the dietary habits of acne patients, assessing the clinical severity of acne, investigating the quality of life of acne patients, and outlining important aspects for future interventional trials.

### 2.4. Statistical Evaluation

A statistician performed all data analyses using SPSS software version 26 (IBM, Armonk, New York, NY, USA). The study population was divided into groups according to ω-3 FA levels: no deficit (HS-omega-3 Index^®^ 8–11%), deficit (HS-omega-3 Index^®^ < 8%), and severe deficit (HS-omega-3 Index^®^ < 4%). Descriptive statistics (mean, median, standard deviation, minimum, and maximum) for demographic data were calculated. Differences between groups were calculated using parametric and non-parametric tests as appropriate. Correlations between patients’ laboratory values and dietary habits were determined using the Mann–Whitney Test. A *p*-value of 0.05 was set as the level of significance for all statistical tests.

## 3. Results

### 3.1. Demographic Data and Acne Severity

The study population included 100 patients (57 females, 43 males) with a mean age of 22 ± 6.9 years and a mean body mass index (BMI) of 25.07 ± 22.0 kg/m^2^. Most patients presented with acne papulopustulosa. Topical prescription medication was reported in 63 patients (adapalen 0.3%/benzoyl peroxide 2.5%, *n* = 18; clindamycin 10 mg/g/benzoyl peroxide 50 mg/g, *n* = 18; adapalen 0.1%/benzoyl peroxide 2.5%, *n* = 13; benzoyl peroxide, *n* = 12; azelaic acid 15–20%, *n* = 11; adapalene 0.1–0.3%, *n* = 8; clindamycin 10 mg/g/tretinoin 0.25 mg/g, *n* = 2), while 22 participants received a systemic prescription treatment (isotretinoin 10–30 mg, *n* = 10; doxycycline 50 mg, *n* = 7; anti-androgen contraceptive, *n* = 9; minocycline 50 mg, *n* = 1). The skin condition had a moderate impact on patients’ quality of life, as evidenced by a mean DLQI score of 6.5 points. Patients’ demographics are presented in Table 1.

### 3.2. Omega-3 Fatty Acid Levels

**Figure 2:**
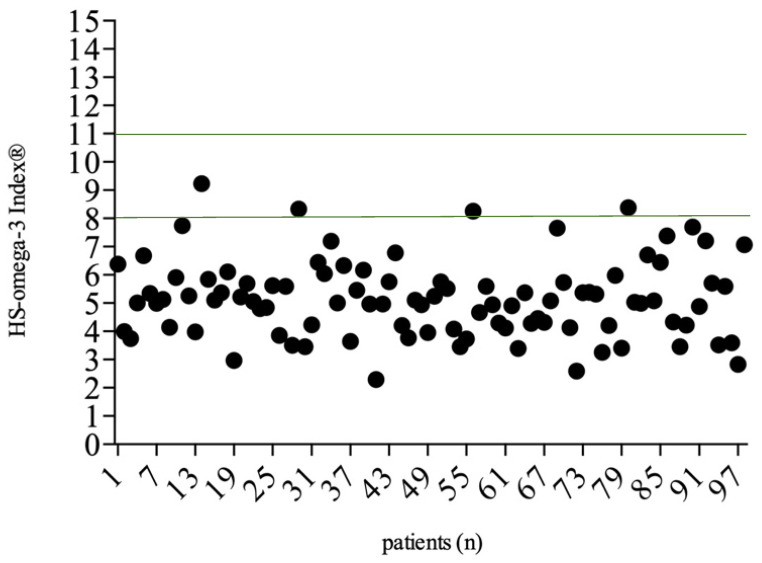
HS Omega-3 index^®^ (%) of investigated acne patients with a recommended range of 8–11% (green line, *n* = 98).

Most acne patients (*n* = 94, 96%) showed a deficit in ω-3 FAs with a mean HS-omega-3 Index^®^ of 5.15%. Overall, the groups were well balanced with respect to the anatomic sites affected and the acne subtype (Table 1, Figure 2). Although not statistically significant, patients without a deficit (*n* = 4) were not affected by the most severe subtype, acne conglobata. Patients under isotretinoin treatment (*n* = 10) showed a lower mean HS-omega-3 Index^®^ of 4.83% compared to the overall mean value of the total population, but not significantly (*p* = 0.347).

Patients with ω-3 FA levels outside the recommended range of 8–11% showed higher IGF-1 levels, compared to patients without a deficit, but not significantly so (311.9 ng/mL vs. 278.25 ng/mL, *p* = 0.424) (Table 2).

When subdividing patients into a severe deficit group (*n* = 20, HS Omega-3 index^®^ < 4%), a male predominance was seen (*p* = 0.021) and IGF-1 levels increased even further (316.48 ng/mL), but not significantly so (r = 0.135, *p* = 0.181) (Table 3). 

### 3.3. Dietary Habits

Five patients were on a dairy-free diet, while no other diets were reported. Correlating the patients’ dietary habits with the respective blood levels revealed that patients who regularly consumed legumes had significantly higher levels of ω-3 FAs than patients who did not consume legumes. (*p* = 0.003) Participants who abstained from sunflower oil had significantly higher levels of ω-3 FAs compared to participants with regular sunflower oil intake. (*p* = 0.008) Acne patients with regular ω-3 FA supplementation had significantly higher ω-3 FA levels compared to patients without ω-3 FA supplementation (*p* = 0.006) (Table 4).

## 4. Discussion

Hippocrates’ dogma of “let food be thy medicine and medicine be thy food” has gained solid evidence in recent years, with clinical trials highlighting the effects of diet on the clinical severity and course of inflammatory dermatoses, including acne [1,32,45]. A negative impact on the management of acne has since been attributed to a Western diet high in saturated fats, highly processed carbohydrates, and dairy products, mainly due to its direct effects on IGF-1 [1,2,35]. However, preventive and therapeutic dietary measures, including the targeted use of nutraceuticals, have not yet been adequately addressed [11,46].

This pilot study provided new insights into ω-3 FA levels in acne patients, with 96% of patients having a HS-omega-3 Index^®^ outside the recommended range of 8–11%. In comparison, in a large cohort of 23,615 volunteers from Europe, 76.15% had a HS-omega-3 Index^®^ below 8% [33]. The mean of 5.15% in the present cohort was also lower compared to a nationwide German cohort of middle-aged women with a mean value of 5.49% [47]. While these data show a general trend towards a ω-3 FA deficit in the general population, our study suggests an exacerbation in acne patients. The reported deficit in EPA and DHA in acne patients provides a basis for future studies of oral ω-3 FA supplementation, as the clinical effects of the targeted supplementation of deficits are expected to be more pronounced than supplementation when target levels are already present [22].

This pilot study categorized patients based on their HS-omega-3 Index^®^ into “no deficit” (HS-omega-3 Index^®^ 8–11%), “deficit” (HS-omega-3 Index^®^ < 8%), and “severe deficit” (HS-omega-3 Index^®^ < 4%). As the majority of acne patients had a deficit, which was unpredictable, the groups were unbalanced after the initial assessment. However, although not significant due to the resulting small number of patients, subdivision showed that the mean HS-omega-3 Index^®^ was even lower in the subgroup of acne patients treated with isotretinoin (4.83%). This may support the hypothesis that isotretinoin-treated patients may specifically benefit from ω-3 FA supplementation, as suggested by previous studies [48,49,50], and this should to be investigated in future trials. In particular, mucocutaneous side effects including cheilitis, xerosis cutis, and dryness of nose and eyes were less frequent in a group that received isotretinoin combined with oral ω-3 FA compared to the group that received isotretinoin alone [51].

Several mechanisms have been discussed for how ω-3 FA may positively influence acne severity [30]. Interestingly, our data suggest a trend towards lower mean ω-3 FA levels and higher mean IGF-1 levels in acne patients. Although not significant, these preliminary data should be investigated in future controlled studies with a larger cohort of patients. It may support the direct effect of ω-3 FAs on IGF-1 and fuel the hypothesis that oral supplementation of ω-3 FAs may directly affect pathophysiological pathways of acne, such as sebum production, by lowering IGF-1 levels [52,53,54]. Although not seen in the present cohort, previous studies have also reported an inverse association between inflammatory biomarkers and ω-3 FA levels [55,56,57].

Very few prospective, interventional human studies have been conducted to investigate the clinical effects of ω-3 FAs on acne, with major limitations in the study designs, including small patient cohorts and short follow-up periods. Remarkably, none of the previous studies investigated the blood levels of ω-3 FAs to establish baseline values. Rubin et al. conducted a study in 2008 on five patients [37]. After eight weeks of supplementation with EPA, combined with micronutrients and a green tea antioxidant, the number of lesions decreased from 62.8 to 40.4 in four out of five patients. There was a reduction in inflammatory lesions count from 20.8 to 6.8. There was also a mean improvement of 24% in patients’ self-rated mental status using a standardized questionnaire. In 2012, Khayef et al. investigated the effects of DHA and docosapentaenoic acid (DPA) in 13 men [38]. In addition to the number of lesions, the authors assessed the patients’ skin redness and evaluated a three-day dietary diary. No significant differences were found when comparing the number of lesions at baseline and 12 weeks after the intervention. However, 8/13 individuals improved, with 7/8 entering the study with moderate to severe lesions. In 4/13 patients, acne severity worsened, with 3/4 patients starting with mild acne at baseline. Jung et al. [31] conducted the highest quality interventional study to date in acne patients, including a histopathologic analysis of facial lesions after 12 weeks of oral ω-3 FA supplementation, which impressively demonstrated reduced inflammatory markers. Furthermore, a recent randomized controlled trial has highlighted the beneficial effects of ω-3 FA supplementation as an adjuvant treatment in acne vulgaris by positively regulating the gut microbiota [58]. In an experimental murine model, increased gut diversity with a raised abundance of butyric acid-producing bacteria was observed after ω-3 FA supplementation. An alleviation of inflammation and comedone count was reported [58]. Future interventional studies in larger cohorts should therefore continue to include clinical assessments such as laboratory profiles, skin biopsies, and gut microbiome sequencing to gain an even deeper understanding of the underlying mechanisms.

The evaluation of patients’ dietary habits revealed that “legume intake” versus “no legume intake” was significantly associated with higher ω-3 FA levels (*p* = 0.003). On the other hand, higher ω-3 FA levels were observed in participants with “no sunflower oil intake” compared to patients with “sunflower oil intake” (*p* = 0.008). Possible explanations may be that legumes provide a high nutritional value of ω-3 FAs, protein, resistant starch, and minerals [59]. Well-known sources include beans, peas, chickpeas, lentils, and lupins. Sunflower oil, on the other hand, is a common source of ω-6 FAs, often found in fried foods and convenience foods [60]. Patients who did not consume sunflower oil may have used other fat sources instead, including rapeseed oil or linseed oil, which have a higher proportion of ω-3 FAs, possibly explaining the higher ω-3 FA levels. Participants who were taking ω-3 FA supplements had significantly higher ω-3 FA levels compared to patients not taking supplements (*p* = 0.006). Baseline levels should be assessed prior to any supplementation, as there is no evidence to suggest any beneficial effects of oversaturation [33].

While preliminary evidence supports the efficacy of ω-3 FA supplementation as an adjunctive treatment for acne patients [49,51], no general clinical recommendation for supplementation can yet be made [30,31,37]. Therefore, we suggest the following for future interventional clinical trials. 1. Baseline ω-3 FA levels of patients should be assessed and participants should be recruited based on the ranges of their ω-3 FA baseline levels. 2. Rather than supplementing with a fixed dose of EPA and DHA, the goal should be to achieve a suggested target range of 8–11% through regular blood level measurements. 3. Since the bioavailability of ω-3 FA supplements can vary widely, as seen in previous studies in cardiac patients [33], patients should take EPA and DHA with the main meal of the day. 4. If clinical effects are found in a cohort with ω-3 FA deficit, further studies including participants regardless of their baseline levels can investigate whether ω-3 FA oversaturation may also have beneficial effects. 5. Finally, dose-finding studies are needed, including assessments of the optimal duration of EPA and DHA supplementation.

As a growing body of evidence points to the impact of so-called exposome factors, including diet, on acne severity [61,62,63], clinicians should be aware of this and be able to address relevant questions from patients. This study adds new insight into feasible lifestyle changes to support acne treatment. The gender- and age-balanced patient cohort with demographics consistent with previous global epidemiologic data of acne patients can be considered a strength of this study [64]. In addition, patients’ dietary habits were assessed using a standardized protocol. Limitations include the single-center design and the lack of an aged-matched control group. Nevertheless, the aim of the present study was to obtain first data on the HS-omega-3 Index^®^ in acne patients. Unfortunately, laboratory parameters of two patients could not be obtained. However, according to the ethical protocol, the clinical assessment of the patients, including dietary habits, was included in the present analysis. Future studies should build on the data extracted from the present trial and investigate the clinical effects in an interventional, controlled design.

## 5. Conclusions

Acne patients showed a deficit in ω-3 FAs regardless of clinical acne severity, with a mean HS-omega-3 Index^®^ of 5.15% (target range 8–11%). A severe deficit (HS-omega-3 Index^®^ < 4%) was more common in men than in women. Legume intake and oral ω-3 FA supplementation were significantly associated with higher levels. Future larger interventional, controlled trials are needed to investigate the potential of oral supplementation in acne patients according to blood levels in erythrocytes.

## Figures and Tables

**Table 1:** Characteristics of the study population.

	Total Cohort (*n* = 100)	ω-3 FA Deficit HS-Omega-3 Index^®^ <8% (*n* = 94/98)	no ω-3 FA Deficit HS-Omega-3 Index^®^ 8–11% (*n* = 4/98)	*p* Value
Females	57 (57%)	51 (54.3%)	4 (100%)	0.131
Males	43 (43%)	43 (45.7%)	0	
Mean age (y)	22.29 (SD = 6.9)	22.14 (SD = 6.9)	25.5 (SD = 8.4)	0.32
BMI (kg/m^2^)	25.07 (SD = 22.0)	25.17 (SD = 22.46)	22.84 (SD = 3.39)	0.895
Acne comedonica	20 (20%)	17 (18.1%)	2 (50%)	0.293
Acne papulopustulosa	70 (70%)	67 (71.3%)	2 (50%)	
Acne conglobata	10 (10%)	10 (10.6%)	0	
Topical treatment	63 (63%)	59 (62.8%)	2 (50%)	0.621
Systemic treatment	22 (22%)	21 (22.3%)	1 (25%)	1

(kg = kilogram; *n* = number; m = meter; y = year; SD = standard deviation).

**Table 2:** Laboratory parameters comparing patients with a deficit (HS Omega-3 index^®^ < 8%) and patients without a deficit.

	Total Cohort (*n* = 100)	ω-3 FA Deficit HS-Omega-3 Index^®^ <8% (*n* = 94/98)	No ω-3 FA Deficit HS-Omega-3 Index^®^ 8–11% (*n* = 4/98)	*p* Value
IGF-1 (ng/mL)	310.53 (SD = 97.7)	311.9 (SD = 98.98)	278.25 (SD = 58.66)	0.424
HbA1_c_ (mmol/mol)	5.17 (SD = 0.35)	5.17 (SD = 0.36)	5.25 (SD = 0.1)	0.552
Fructosamin (µmol/L)	268.06 (SD = 20.63)	267.99 (SD = 20.9)	269.75 (SD = 14.73)	0.536
HDL (mg/dL)	56.92 (SD = 12.92)	56.6 (SD = 13.0)	64.5 (SD = 9.26)	0.205
LDL (mg/dL)	96.44 (SD = 27.86)	96.14 (SD = 27.66)	103.5 (SD = 36.13)	0.808
Triglycerides (mg/dL)	95.05 (SD = 49.94)	95.49 (SD = 50.74)	84.75 (SD = 26.04)	0.936
Insulin	21.45 (SD = 27.0)	21.57 (SD = 27.5)	18.7 (SD = 11.7)	0.553
CRP (mg/dL)	0.21 (SD = 0.4)	0.2 (SD = 0.4)	0.32 (SD = 0.46)	0.853
Leukocytes (G/L)	7.2 (SD = 1.8)	7.22 (SD = 1.83)	6.6 (SD = 0.9)	0.468

(dL = deciliter; G = giga; L = liter; mg = milligram; mmol = millimole; *n* = number; ng = nanogram; SD = standard deviation).

**Table 3:** Patients’ demographics and laboratory parameters comparing participants with a deficit and severe ω-3 FA deficit.

	ω-3 FA Deficit HS-Omega-3 Index^®^ <8% (*n* = 74/94)	Severe ω-3 FA Deficit HS-Omega-3 Index^®^ <4% (*n* = 20/94)	*p* Value
Females	45 (60.8%)	6 (30.0%)	0.021
Males	29 (39.2%)	14 (70%)	
Mean age (y)	22.63 (SD = 7.28)	20.45 (SD = 5.14)	0.215
BMI (kg/m^2^)	25.9 (SD = 25.5)	22.5 (SD = 2.8)	0.895
Acne comedonica	18 (24.3%)	3 (15%)	0.521
Acne papulopustulosa	51 (68.9%)	14 (70%)	
Acne conglobata	5 (6.8%)	3 (15%)	
IGF-1 (ng/mL)	310.59 (SD = 104.4)	316.48 (SD = 79.45)	0.812
HbA1_c_ (mmol/mol)	5.14 (SD = 0.32)	5.28 (SD = 0.32)	0.16
Fructosamin (µmol/L)	270.23 (SD = 21.18)	260.19 (SD = 18.27)	0.052
HDL (mg/dL)	57.96 (SD = 13.13)	51.86 (SD = 11.60)	0.058
LDL (mg/dL)	98.99 (SD = 28.66)	86.24 (SD = 21.67)	0.062
Triglycerides (mg/dL)	95.23 (SD = 45.11)	96.38 (SD = 68.15)	0.928
Insulin	21.69 (SD = 26.5)	21.14 (SD = 31.22)	0.936
CRP (mg/dL)	0.21 (SD = 0.45)	0.20 (SD = 0.24)	0.905
Leukocytes (G/L)	7.21 (SD = 1.85)	7.24 (SD = 1.76)	0.96

(dL = deciliter; G = giga; L = liter; mg = milligram; mmol = millimole; *n* = number; ng = nanogram; SD = standard deviation; y = year).

**Table 4:** Significant correlations of patients’ dietary habits and median ω-3 FA values (*n = number*).

	Median HS-Omega-3 Index^®^ % (*n*) Consumption of….	Median HS-Omega-3 Index^®^ % (*n*) No Consumption of…	*p* Value
Legumes	5.36 (70)	4.33 (23)	0.003
ω-3 FA supplement	5.69 (13)	4.98 (82)	0.006
Sunflower oil	4.95 (69)	5.38 (25)	0.008

## Data Availability

The data presented in this study are available on request from the corresponding author. The data are not publicly available due to privacy restrictions.
